# The Implications of Lineage-Specific Rates for Divergence Time Estimation

**DOI:** 10.1093/sysbio/syz080

**Published:** 2019-12-06

**Authors:** Tom Carruthers, Michael J Sanderson, Robert W Scotland

**Affiliations:** 1 Department of Plant Sciences, University of Oxford, South Parks Road, Oxford OX1 3RB, UK; 2 Department of Ecology and Evolutionary Biology, University of Arizona, 1041 East Lowell, Tucson, AZ 85721-0088, USA

## Abstract

Rate variation adds considerable complexity to divergence time estimation in molecular phylogenies. Here, we evaluate the impact of *lineage-specific rates*—which we define as among-branch-rate-variation that acts consistently across the entire genome. We compare its impact to *residual rates*—defined as among-branch-rate-variation that shows a different pattern of rate variation at each sampled locus, and *gene-specific rates*—defined as variation in the average rate across all branches at each sampled locus. We show that lineage-specific rates lead to erroneous divergence time estimates, regardless of how many loci are sampled. Further, we show that stronger lineage-specific rates lead to increasing error. This contrasts to residual rates and gene-specific rates, where sampling more loci significantly reduces error. If divergence times are inferred in a Bayesian framework, we highlight that error caused by lineage-specific rates significantly reduces the probability that the 95% highest posterior density includes the correct value, and leads to sensitivity to the prior. Use of a more complex rate prior—which has recently been proposed to model rate variation more accurately—does not affect these conclusions. Finally, we show that the scale of lineage-specific rates used in our simulation experiments is comparable to that of an empirical data set for the angiosperm genus *Ipomoea*. Taken together, our findings demonstrate that lineage-specific rates cause error in divergence time estimates, and that this error is not overcome by analyzing genomic scale multilocus data sets. [Divergence time estimation; error; rate variation.]

Since the proposal of the “molecular clock” hypothesis, which made the important assumption that differences between homologous sequences accumulate at a constant rate over time (Zuckerkandl and Pauling 1962, 1965), there has been a continual interest in estimating divergence times in molecular phylogenies (Hori and Osawa 1979; Miyata et al. 1980; Kumar and Hedges 1998; Aris-Brosou and Yang 2003; Kumar 2005). Often, the key assumption of the “molecular clock” is violated and evolutionary rates differ between branches in a phylogeny (Langley and Fitch 1974; Britten 1984; Gillespie 1989, 1991; Bromham et al. 1996). This can fundamentally compromise divergence time estimates, even when using methods that incorporate rate variation. This is because the number of substitutions along any particular branch—the parameter directly inferred from molecular sequence data—is a product of the rate of molecular evolution and the branch’s temporal duration (Gillespie 1991; Sanderson 1997, 2002; Thorne et al. 1998; Britton 2005). Without making assumptions about rate variation or divergence times, distinguishing models with different patterns of rate variation or branch duration can therefore become an intractable problem (Sanderson 1997, 2002; Thorne et al. 1998; Kishino et al. 2001).

Previous studies have evaluated the impact of among-branch-rate-variation on divergence time estimates and presented new methodologies to account for its effects (Sanderson 1997, 2002; Drummond et al. 2006; Smith and O’Meara 2012; Tamura 2012). These studies have provided a fundamental basis for understanding how different assumptions about among-branch-rate-variation affect divergence time estimates. In contrast to these studies focusing on specific methodologies, Britton (2005) provided a more general analysis of the implications of among-branch-rate-variation for divergence time estimation. Using a mathematical model in the context of a three-taxon tree, Britton (2005) demonstrated that among-branch-rate-variation leads to erroneous divergence time estimates, regardless of the length of molecular sequence analyzed, whether likelihood or Bayesian inference was used, and even when the correct model of among-branch-rate-variation was used. Britton (2005) succinctly highlighted that this error results from the fact that molecular sequence data does not provide information about rates for individual branches.

With increasingly large molecular data sets, in which multiple fossil calibrations may also be implemented, sources of uncertainty in divergence time estimates can become increasingly complex. Several studies have attempted to investigate these sources of uncertainty. Yang and Rannala (2006), Rannala and Yang (2007), and dos Reis and Yang (2013) have described how uncertainty results from either limited molecular sequence data or uncertain fossil calibrations. As such, when a large amount of molecular data is sampled, they conclude that uncertainty stems almost exclusively from fossil calibrations. Although this framework was developed according to the assumptions of the “molecular clock,” they have indicated that it is applicable when there is among-branch-rate-variation, provided a large number of loci are sampled (Rannala and Yang 2007; Zhu et al. 2015). However, when accounting for among-branch-rate-variation, they assume it shows different patterns at each sampled locus, rather than acting consistently across the entire genome (Rannala and Yang 2007; Zhu et al. 2015). Ho (2014) has stated that these different interpretations of among-branch-rate-variation will have important effects on divergence time estimates, but no analyses have been undertaken to characterize these effects in different contexts.

Taken together, previous studies have illustrated the important effects of among-branch-rate-variation on divergence time estimates, and that these effects can become increasingly complex in genomic scale data sets. However, current understanding of the implications of rate variation in genomic scale data sets does not fully take into account many of the more complex ways in which rates can vary in these data sets, and the effects this can have on divergence time estimates.

Here, we perform simulation experiments to evaluate the extent to which among-branch-rate-variation that acts consistently across entire genomes leads to error in divergence time estimates. We refer to this class of rate variation as *lineage-specific rates* (Fig. 1a). We do not use the term *lineage effects* because Gillespie (1989) originally used this term to describe the effect of rate and time on the number of substitutions. We compare the implications of lineage-specific rates to those of *gene-specific rates*—where the average rate across all branches varies at each sampled locus (Fig. 1b), and *residual rates*—where rates vary between branches, but the pattern of among-branch-rate-variation is different at each sampled locus (Fig. 1c). We also evaluate the implications when more than one class of rate variation occurs simultaneously (Fig. 1d).

**Figure 1.**
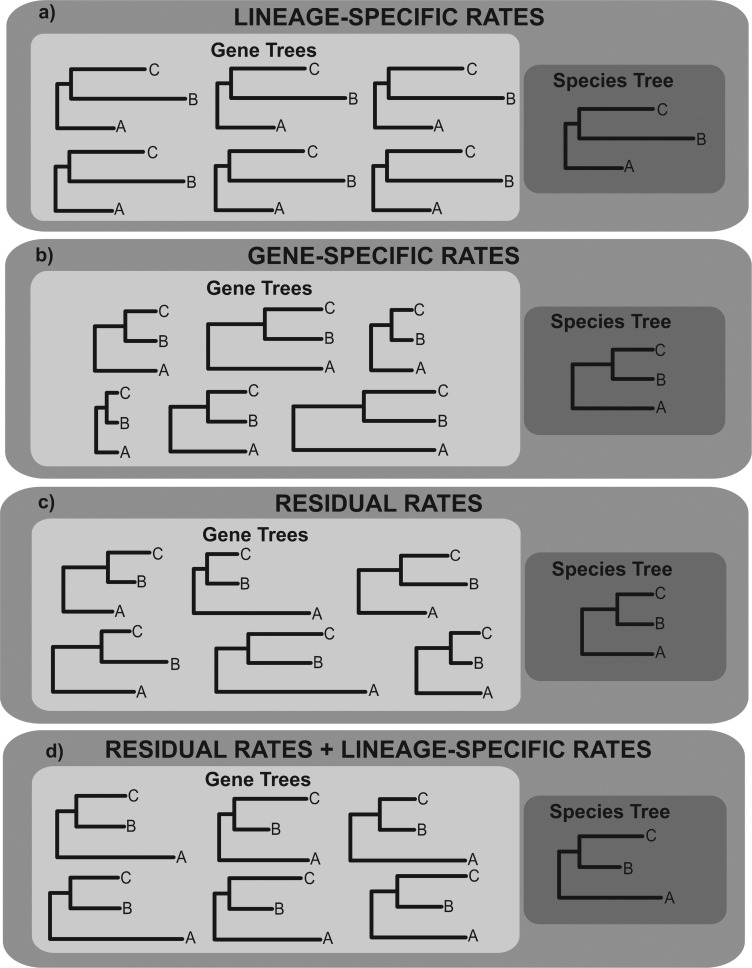
A summary of different classes of molecular evolutionary rate variation. The trees shown are phylograms, and in all cases taxa are sampled at the present. In all trees, the two divergence events occurred at time $x$ in the past, and time $2x$ in the past. Therefore, branch length variation reflects molecular evolutionary rate variation. a) Lineage-specific rates. Among-branch-rate-variation is observed in each gene tree, but the pattern of among-branch-rate-variation is the same for each gene tree. The average rate for each branch therefore differs in the species tree. b) Gene-specific rates. The average rate across all branches differs for each gene tree but does so consistently for all branches. The average rate for each branch is therefore the same in the species tree. c) Residual rates. Among-branch-rate-variation is observed in all gene trees, but the pattern of among-branch-rate-variation is different for each gene tree. If a large number of loci are sampled, the average rate for each branch is the same in the species tree. d) Residual rates and lineage-specific rates. Because of the underlying lineage-specific rate, the average rate for each branch differs in the species tree.

We assess how these classes of rate variation influence divergence time estimates when different quantities of molecular data are analyzed. We alter the quantity of molecular data by either changing the number of loci, or by changing the sequence length at a single locus. We also compare the performance of two “relaxed clock” priors—one of which has been proposed as particularly effective for divergence time estimation when analyzing multi-locus data sets that exhibit rate variation (dos Reis et al. 2014). Finally, we analyze a phylogeny of the angiosperm genus *Ipomoea* to determine whether the lineage-specific rates simulated in our experiments are of a comparable magnitude to those that occur in real data sets.

The issues dealt with in this article have significant implications for divergence time estimation because there is widespread evidence that lineage-specific rates are prevalent in many groups, and that they can significantly bias divergence time estimates (Langley and Fitch 1974; Britten 1984; Gillespie 1989, 1991; Bromham et al. 1996; Phillips and Fruciano 2018). Lineage-specific rates have been associated with a range of variables including generation time, efficiency of DNA repair mechanisms, metabolic rates, growth form, or whether the organism is free living or parasitic (Gillespie 1989, 1991; Duff and Nickrent 1997; Lanfear et al. 2013; Ho 2014).

## Materials and Methods

### Summarizing the effects of different classes of rate variation

Most simulations were centered on a three-taxon species tree, with a root age of 1.0, and an age for the single internal node of 0.5. To simulate lineage-specific rates, we multiplied each branch time duration of this species tree by a different lineage-specific rate drawn from a lognormal distribution (mean $[\mu] = -0.01$, standard deviation $[\sigma] = 0.15)$. This generates a species tree with branch lengths that reflect lineage-specific rates. We then simulated 400 gene trees that are identical to this species tree. At this stage, the gene trees are identical to the species tree because gene-specific rates and residual rates have not yet been simulated. To simulate gene-specific rates, we multiplied all branch lengths in each gene tree by a gene-specific rate drawn from a lognormal distribution ($\mu = -0.01, \sigma = 0.15$). A new gene-specific rate was drawn for each gene tree. To simulate residual rates, we multiplied each branch length in each gene tree by a residual rate drawn from a lognormal distribution $(\mu = -0.01, \sigma = 0.15)$. In these experiments, we generated gene trees with; no lineage-specific rates, gene-specific rates, or residual rates; only lineage-specific rates; only gene-specific rates; only residual rates; and residual rates and lineage-specific rates. Different classes of rate variation were simulated with a custom R script that required the packages phytools (Revell 2012, 2017) and phylobase (Bolker et al. 2017).

DNA sequences of 800 base pairs (bp) were simulated along the branches of each gene tree with a custom R script that used the simSeq() function from the package phangorn (Schliep 2011, 2018). Sequences were simulated with a JC model with rate set to 0.05. Simulated sequences were then compiled into one of three data sets. One contained a sequence from a single locus, the second contained sequences from 20 concatenated loci (a total of 16,000 bp), and the third contained sequences from all 400 concatenated loci (a total of 320,000 bp). For each experiment, we also simulated two further data sets for a single locus of different lengths: one 16,000 bp data set and one 320,000 bp data set.

We inferred the divergence time of the single internal node in the three-taxon tree using RevBayes (Hohna et al. 2016). The topology was fixed to that of the initial species tree, and the root age was fixed at 1.0. The “correct” value for the single unknown divergence time—the internal node—is 0.5. The prior on the branching process was a pure birth (Yule) model. The speciation rate $(\lambda)$ was sampled from an exponentially distributed prior with a rate parameter of 10.0. Analyses run without sequence data using this prior produced a posterior divergence time that was uniformly distributed between 0.0 and 1.0. Our results are therefore unlikely to be influenced by the time prior defined by this branching process. We used two different models for the prior on molecular evolutionary rates. A strict clock fixed the rate at 0.05 for all four branches in the three-taxon tree and for all sampled loci. A relaxed clock prior used an uncorrelated lognormal (UCLN) relaxed clock $(\mu = -3.01, \sigma = 0.15)$, in which a separate rate was inferred for each branch in the three-taxon tree, but the same rate was used across all sampled loci. We performed 200 replicates of the entire experiment outlined above.

The UCLN relaxed clock used in these experiments may be a poor fit to data that has been simulated with gene-specific rates. We therefore performed additional experiments using a prior which more explicitly accounts for gene-specific rates. This ensured that none of the conclusions made were misleading (Supplementary Appendix S1 available on Dryad at http://dx.doi.org/10.5061/dryad.m1n6m0m).

### Increasing the strength of lineage-specific rates

We performed a further experiment where sequences were simulated with lineage-specific rates of different strengths (from lognormal distributions with $\sigma = 0$, 0.15, 0.3, or 0.6). These sequences were also simulated with residual rates, with the same parameters as previously. We inferred the unknown divergence time in RevBayes (Hohna et al. 2016) using two different UCLN relaxed clocks. In one, $\sigma$ of the UCLN relaxed clock was fixed at 0.15, whilst in the other, $\sigma$ of the UCLN relaxed clock was altered to exactly match $\sigma$ of the distribution from which lineage-specific rates had been simulated. When inferring the unknown divergence time, all 400 simulated loci were analyzed.

### Comparing two different priors for rate heterogeneity

In the above experiments, the UCLN relaxed clock infers a single branch specific rate across all sampled loci. Although this is a widely used approach, we also evaluated the performance of an alternative prior—the Dirichlet rate prior—which has been suggested for multilocus data sets that exhibit rate variation (dos Reis et al. 2014; Zhu et al. 2015).

This prior uses a gamma or lognormal distribution for the mean rate amongst all sampled loci ($\bar{\mu}$). With being the number of sampled loci, a Dirichlet distribution then partitions the total rate ($\bar{\mu}^* L$) amongst each of the sampled loci to infer a mean rate for each locus. For each locus, different rates are then inferred for each branch with a UCLN relaxed clock that is specific to that locus and parameterized with the locus specific mean.

We compared the performance of the Dirichlet rate prior to the UCLN relaxed clock used previously. The Dirichlet rate prior was parameterized as follows: $\bar{\mu}$ was sampled from a lognormal distribution ($\mu = -3.01, \sigma = 0.15$), the concentration parameter for the Dirichlet distribution of locus specific rates was 1, and the UCLN model applied to each locus was parameterized with $\mu = -3.01$ and $\sigma = 0.15$. The UCLN relaxed clock that inferred a single branch specific rate across all loci was parameterized as previously ($\mu = -3.01, \sigma = 0.15$). We characterized the performance of these priors when there were lineage-specific rates, gene-specific rates, and residual rates. We simulated these classes of rate variation as described previously. The unknown divergence time was inferred in RevBayes (Hohna et al. 2016), using all 400 simulated loci.

### Evaluating lineage-specific rates in eight-taxon trees

We evaluated the implications of lineage-specific rates in eight-taxon trees to determine whether our findings from experiments with three-taxon trees are likely to be applicable in larger trees. We used a custom R script that used the function sim.bd.taxa() from the package TreeSim to simulate trees. Trees were simulated with $\lambda = 1$, $\mu = 0$, and the root age was unfixed. Gene trees, rate variation, and DNA sequences were simulated according the same principles as with the three-taxon trees. We simulated DNA sequences with no rate variation, or lineage-specific rates from a lognormal distribution with $\mu = -3.01$ and $\sigma = 0.15$.

We inferred divergence times using RevBayes (Hohna et al. 2016). The topology was fixed to that of the initial species tree and the root age was fixed to the correct value. The prior for the branching process was a pure birth (Yule) model with $\lambda $ fixed to 1 (the correct value). Unlike with the three-taxon trees, analyses run without molecular sequence data produced posterior divergence time estimates that were not uniformly distributed between 0.0 and the root age. Therefore, in this analysis, inferred divergence times are likely to be influenced by the branching process. This analysis therefore provides a less direct illustration of the effects of rate variation, but allows its effects to be investigated in a more complex tree. We used two different models for the prior on molecular evolutionary rates. A strict clock fixed the rate at 0.05 for all branches and for all sampled loci. A relaxed clock prior used an UCLN relaxed clock ($\mu = -3.01, \sigma = 0.15$), in which a separate rate was inferred for each branch, but the same rate was used across all sampled loci.

Custom R, Revbayes, and Python scripts that were developed for all simulation experiments are available in Supplementary Materials available on Dryad. Simulated matrices and other output files are available on request.

### An empirical study: quantifying the magnitude of lineage-specific rates in Ipomoea

We quantified the magnitude of lineage-specific rates in a phylogeny for *Ipomoea* that was inferred from a dataset 434 concatenated nuclear genes (Muñoz-Rodríguez et al. 2019). We extracted all 76 pairs of sister species (terminal taxa) in this phylogeny. Because these sister pairs have the same branch time duration, by definition, they can provide some insight into the extent of lineage-specific rates. We could therefore evaluate whether the lineage-specific rates simulated in our experiments are of a comparable scale to those that occur in a biological data set.

## Results

### Summarizing the effects of different classes of rate variation

In the results presented in detail here, the UCLN relaxed clock was used to infer the unknown divergence time. A comparison with the results obtained when using the strict clock follows.

#### No rate variation.

**Figure 2.**
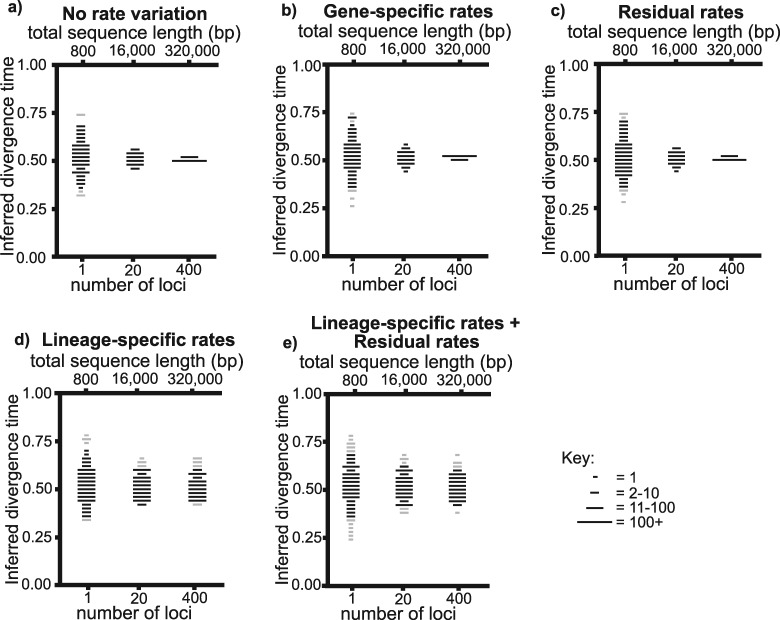
Summary of results from simulation experiments with different classes of rate variation and different numbers of sampled loci (each locus has a length of 800 bp). The unknown divergence time is inferred with a UCLN relaxed clock. Mean posterior age estimates from each experiment are plotted within bins of 0.2. The length of each plotted line corresponds to the number of estimates within each bin (see key). The line is plotted in gray if for more than 50% of replicate experiments plotted within a bin, the 95% HPD did not include the correct value. Otherwise the line is plotted in black. a) No rate variation, b) gene-specific rates, c) residual rates, d) lineage-specific rates, and d) lineage-specific rates and residual rates.

Increasing the number of sampled loci led to reduced error in mean posterior age estimates (Fig. 2a). The root mean squared error (RMSE)—which quantifies the magnitude of error in the same units as the simulation experiment—fell from 0.0791 when sampling 1 locus, to 0.0108 when sampling 400 loci (Supplementary Table S1 available on Dryad). Increasing the number of sampled loci also caused the mean 95% highest posterior density (HPD) width to decrease from 0.346 to 0.192, and the percentage of replicate experiments that included the correct value in the 95% HPD to increase from 97.5% to 100% (Supplementary Table S1 available on Dryad). Increasing the sampled sequence length at a single locus had an indistinguishable effect from increasing the number of sampled loci (Supplementary Fig. S1a, Table S1 available on Dryad).

#### Gene-specific rates.

Increasing the number of sampled loci led to reduced error in mean posterior age estimates (Fig. 2b). The RMSE fell from 0.0818 when sampling 1 locus to 0.0114 when sampling 400 loci (Supplementary Table S1 available on Dryad). The mean 95% HPD width, and percentage of replicate experiments that included the correct value within the 95% HDP, were similar to when there was no rate variation (Supplementary Table S1 available on Dryad).

When the sampled sequence length at a single locus was increased, the reduction in error was considerably less (Supplementary Fig. S1b available on Dryad). The RMSE fell from 0.0818 when sampling 800 bp to 0.0457 when sampling 320,000 bp (Supplementary Table S1 available on Dryad). The mean 95% HPD widths were similar to when incrementally more loci were sampled (Supplementary Table S1 available on Dryad), whilst fewer replicate experiments included the correct value within the 95% HPD (Supplementary Table S1 available on Dryad). When a prior that more explicitly accounts for gene-specific rates was used, and 320,000 bp were sampled from a single locus, the RMSE fell such that it more closely resembled the case where 400 loci were sampled (Supplementary Appendix S1 and Table S2 available on Dryad).

#### Residual rates.

Increasing the number of sampled loci led to reduced error in mean posterior age estimates (Fig. 2c). The RMSE fell from 0.0886 when sampling 1 locus, to 0.0106 when sampling 400 loci (Supplementary Table S1 available on Dryad). The mean 95% HPD widths, and percentage of replicate experiments that included the correct value within the 95% HPD, were similar to previous experiments in which the number of sampled loci was incrementally increased (Supplementary Table S1 available on Dryad).

When the sequence length at a single locus was increased, the reduction in error was considerably less (Supplementary Fig. S1c available on Dryad). The RMSE fell from 0.0886 when sampling 800 bp to 0.0486 when sampling 320,000 bp (Supplementary Table S1 available on Dryad). The mean 95% HPD widths were similar to when the number of sampled loci was incrementally increased, whilst fewer replicate experiments included the correct value within the 95% HPD (Supplementary Table S1 available on Dryad).

#### Lineage-specific rates.

Increasing the number of sampled loci had a limited impact with respect to reducing error in mean posterior age estimates (Fig. 2d). The RMSE fell from 0.0875 when sampling 1 locus, to 0.0510 when sampling 400 loci (Supplementary Table S1 available on Dryad). The mean 95% HPD widths were similar to previous experiments (Supplementary Table S1 available on Dryad), whilst the percentage of replicate experiments that included the correct value within the 95% HPD was lower than previous experiments in which the number of sampled loci was incrementally increased. Increasing the sampled sequence length at a single locus had a very similar impact to sampling more loci (Supplementary Fig. S1d and Table S1 available on Dryad).

#### Lineage-specific rates and residual rates.

Increasing the number of sampled loci had a moderate impact with respect to reducing error in mean posterior age estimates (Fig. 2e). The RMSE fell from 0.108 when sampling 1 locus, to 0.0518 when sampling 400 loci (Supplementary Table S1 available on Dryad). Mean 95% HPD widths were similar to previous experiments, whilst the percentage of replicate experiments that included the correct value within the 95% HDP increased from 87.5% when sampling 1 locus to 95.5% when sampling 400 loci (Supplementary Table S1 available on Dryad).

Increasing the sampled sequence length at a single locus had a more limited impact with respect to reducing error in mean posterior age estimates (Supplementary Fig. S1e). The RMSE fell from 0.108 when sampling 800 bp to 0.0743 when sampling 320,000 bp. Mean 95% HPD widths were similar to previous experiments. The percentage of replicate experiments that included the correct value within the 95% HPD fell from 87% when sampling 800 bp to 80.5% when sampling 320,000 bp (Supplementary Table S1 available on Dryad).

#### Using a strict clock to infer the unknown divergence time.

The distribution of mean posterior age estimates was broadly the same when the unknown divergence time was inferred with the strict clock compared to the UCLN relaxed clock (Supplementary Table S3 available on Dryad). However, the mean 95% HPD widths were far narrower. As such, the correct value was included within the 95% HPD in far fewer replicate experiments. This is most notably the case when lineage-specific rates were present and the largest molecular data sets were sampled (either 400 loci, or 320,000 bp at a single locus). In these instances, the 95% HPD included the correct value only about 10% of the time (Supplementary Table S3 available on Dryad).

### Stronger lineage-specific rates lead to larger error in divergence time estimates

**Figure 3.**
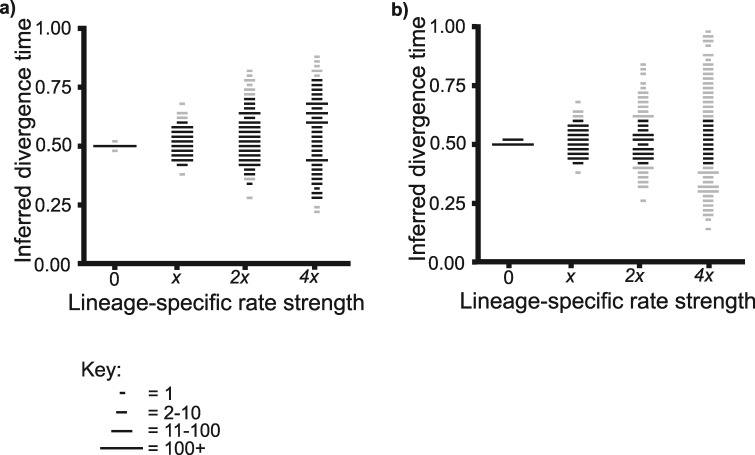
The impact of increasingly strong lineage-specific rates on divergence time estimates. Points are plotted according to the same bins, sizes, and colors as Figure 2. In all cases, the unknown divergence time is inferred from 400 loci. a) $\sigma$ of the UCLN relaxed clock is corrected such that it exactly matches $\sigma$ of the distribution from which lineage-specific rates are simulated. b) $\sigma$ of the UCLN relaxed clock is fixed at 0.15.

Stronger lineage-specific rates caused mean posterior age estimates to differ more from the correct value. This was the case regardless of whether $\sigma$ of the UCLN relaxed clock was corrected such that it was equal to $\sigma$ of the distribution from which lineage-specific rates were simulated (Fig. 3a,b). When $\sigma$ of the UCLN relaxed clock was corrected, the RMSE increased from 0.00363 when there were no lineage-specific rates, to 0.150 with the strongest lineage-specific rates (Supplementary Table S4 available on Dryad). When $\sigma$ was fixed at 0.15, the RMSE increased from 0.0108 to 0.195 (Supplementary Table S4 available on Dryad).

When $\sigma$ was corrected, the mean 95% HPD width increased from 0.0145 with no lineage-specific rates, to 0.588 with the strongest lineage-specific rates. The percentage of replicate experiments that included the correct value within the 95% HPD showed only a small fall from 94% with no lineage-specific rates, to 92.5% with the strongest lineage-specific rates (Supplementary Table S4 available on Dryad). When $\sigma$ was fixed at 0.15, the mean 95% HPD width initially remained constant at around 0.192, but then showed a slight fall to around 0.176 with the strongest lineage-specific rates (Supplementary Table S4 available on Dryad). The percentage of replicate experiments that included the correct value within the 95% HPD decreased from 100% with no lineage-specific rates to 32% with the strongest lineage-specific rates (Supplementary Table S4 available on Dryad).

### The Dirichlet rate prior does not reduce error when there are lineage-specific rates

The Dirichlet rate prior did not reduce error in mean posterior age estimates. The RMSE for the Dirichlet rate prior was 0.0599, whilst the RMSE for the UCLN relaxed clock was 0.0552 (Supplementary Table S5 available on Dryad). The Dirichlet rate prior did result in considerably narrower 95% HPD widths. For the Dirichlet rate prior, the mean 95% HPD width was 0.0186, whilst for the UCLN relaxed clock, the mean 95% HDP width was 0.190. The Dirichlet rate prior only included the correct value within the 95% HPD in 10% of replicate experiments, whilst the UCLN relaxed clock included the correct value within the 95% HPD in 89% of replicate experiments.

### Error resulting from lineage-specific rates is not specific to three-taxon trees

In this section, the correct divergence time is not fixed at 0.5. Therefore, mean posterior estimates (MPEs) are evaluated with respect to the percentage error relative to the correct value, and 95% HPDs are evaluated with respect to their width relative to the MPE.

Lineage-specific rates led to erroneous divergence time estimates in eight-taxon trees. When a UCLN relaxed clock was used and 400 loci were sampled, the mean error for the MPE was 7.785% when there were lineage-specific rates, and was 1.421% when there were no lineage-specific rates (Supplementary Table S6 available on Dryad). Mean 95% HPD widths were 29.510% of the MPE when there were lineage-specific rates, and 29.006% of the MPE when there were no lineage-specific rates. The percentage of nodes for which the 95% HDP contained the correct value was 88.9% when there were lineage-specific rates and was 100% when there were no lineage-specific rates. When a strict clock was used, the mean error for the MPE was similar, but the 95% HPDs were far narrower and the percentage of nodes for which the 95% HPD contained the correct value was far lower (Supplementary Table S6 available on Dryad).

### Lineage-specific rates in Ipomoea are of a comparable scale to those used in our simulation experiments

Across all sister species comparisons in our *Ipomoea* species tree, there is a mean rate difference of 1.23-fold. This implies stronger lineage-specific rates than the majority of our simulation experiments in which rates were drawn from a lognormal distribution with $\sigma = 0.15$. Such a distribution results in a mean rate difference between sister species of 1.19-fold.

## Discussion

### Lineage-specific rates cause divergence time estimation error

Lineage-specific rates lead to error in divergence time estimates, and increasing the number of sampled loci, or sampled sequence length at a single locus, has a limited effect in reducing this error (Fig. 2d,e, and Supplementary Table S1 available on Dryad). For example, when lineage-specific rates were present, increasing the number of sampled loci from 1 to 400 only caused a decrease in error from 17.5% to 10.2% (percentage errors represent the RMSE of mean posterior age estimates as a percentage of the correct value). By contrast, with residual rates, increasing the number of sampled loci from 1 to 400 caused error to decrease from 17.7% to 2.21%. Moreover, increasingly strong lineage-specific rates lead to increasingly large errors in age estimates (Fig. 3a,b, and Supplementary Table S4 available on Dryad). These results are not specific to the case of the three-taxon tree that we used in most experiments, because lineage-specific rates led to a similar level of error in our additional experiments with eight-taxon trees (Supplementary Table S6 available on Dryad). The slightly reduced error in the eight-taxon trees is likely to be caused by the tree prior which in our simulations is correctly parameterized such that is consistent with the parameters under which the trees were simulated. This is unlikely to be the case in an empirical data set. A further important result is that the error caused by lineage-specific rates is not reduced by using a more complex rate prior that has been specifically developed for multilocus data sets. Instead, this more complex prior led to misleadingly precise divergence time estimates (Supplementary Table S5 available on Dryad).

### Comparing different classes of rate variation reveals why lineage-specific rates are problematic

Increasing the quantity of molecular sequence data enables the relative number of substitutions along each branch to be inferred with less error (Britton 2005). With no rate variation, such that the substitution rate is the same on every branch, this means that the unknown divergence time is inferred with less error (Fig. 2a, Supplementary Fig. S1a, Tables S1 and S3 available on Dryad). Further, with no rate variation, and therefore no rate differences between loci, the effect of increasing the sequence length at a single locus is effectively the same as increasing the number of sampled loci (Fig. 2a, Supplementary Fig. S1a, Tables S1 and S3 available on Dryad).

With locus specific classes of rate variation (gene-specific rates and residual rates), increasing the number of sampled loci means that the average substitution rate of sampled loci more accurately reflects that of the entire genome. When there are gene-specific rates, this means that unusually fast or slow loci are less likely to cause poor model parameterization and error in divergence time estimates (Fig. 2b, Supplementary Tables S1 and S3, Appendix S1, available on Dryad). When there are residual rates, this means that the problem reduces to a “strict” molecular clock because there are no overall rate differences among branches across the entire genome (Fig. 1c)—this in turn leads to less error in inferred divergence times (Fig. 2c, Supplementary Tables S1 and S3 available on Dryad). Alternatively, when only the sequence length at a single locus is increased, an average rate across the entire genome cannot be accurately inferred. As such, for both gene-specific rates and residual rates, the reduction in error is far more limited (Supplementary Fig. S1b,c, Tables S1 and S3 available on Dryad)

For lineage-specific rates, however, sampling more loci does not lead to the same improvements in accuracy as are possible for residual rates. In this case, the average substitution rate across all sampled loci is different for each branch, regardless of the number of loci that are sampled (Fig. 1d). Because of this, sampling more loci has a far more limited effect with respect to reducing error in divergence time estimates, and is no more effective than increasing the sampled sequence length at a single locus (Fig. 2d, Supplementary Fig S1d, Tables S1 and S3 available on Dryad).

When there are residual rates and lineage-specific rates, sampling more loci is marginally more effective at reducing error than increasing the sequence length at a single locus (Fig. 2e, Supplementary Fig. S1e, Tables S1 and S3 available on Dryad). This may be because increasing the number of sampled loci reduces error associated with residual rates, whilst error resulting from lineage-specific rates remains.

### Interpreting error in the context of 95% HPD intervals

Our discussion of error has so far focused on mean posterior age estimates. This provides a useful framework to evaluate the effect of different classes of rate variation and different quantities of molecular sequence data. However, when inferred in a Bayesian framework, divergence time estimates are often discussed with respect to the 95% HPD.

In our study, the 95% HPD width is sensitive to the quantity of data that is analyzed (increasing the number of sampled loci and the sequence length at a single locus have an identical effect), and the rate prior that is used. The class of rate variation with which sequences were simulated did not affect the 95% HPD width—except potentially for very strong lineage-specific rates (Supplementary Tables S1, S3, and S4 available on Dryad).

In our initial experiments that evaluated different classes of rate variation, and where the unknown divergence time was inferred with a UCLN relaxed clock, the variance of the UCLN relaxed clock was equal to the variance of the distributions from which rate variation was simulated. Thus, when a single class of rate variation was simulated, a high percentage (approximately 95% or more) of replicate experiments included the correct value within the 95% HPD (Fig. 2, Supplementary Fig S1, Table S1 available on Dryad).

However, when there was more than one class of rate variation (lineage-specific rates and residual rates), and 320,000 bp from a single locus were sampled, only 80.5% of replicate experiments included the correct value within the 95% HPD (Supplementary Fig. S1e and Table S1 available on Dryad). In this case, the variance of the UCLN relaxed clock appears to have been insufficient to account for both classes of simulated rate variation. This contrasts to the case where 400 loci were sampled, and lineage-specific rates and residual rates were present. In this case, 94.5% of replicate experiments included the correct value within the 95% HPD. Here, sampling more loci is likely to have reduced the impact of residual rates. As such, the variance of the UCLN relaxed clock was sufficient to account for remaining rate variation that stemmed from lineage-specific rates (Fig. 2e and Supplementary Table S1 available on Dryad).

Two further cases highlight that with lineage-specific rates, the utility of the 95% HPD is highly sensitive to the variance of the rate prior, even when the maximum number of loci is sampled. First, when strong lineage-specific rates were simulated, and the variance of the UCLN relaxed clock was fixed at a low value, only 32% of replicate experiments included the correct value within the 95% HPD (Fig. 3b, Supplementary Table S4 available on Dryad). Second, when the unknown divergence time was inferred with a strict clock, lineage-specific rates were present, and 400 loci were sampled, only 11.5% of replicate experiments included the correct value within the 95% HPD (Supplementary Table S3 available on Dryad).

Given that the rate prior is rarely well informed, the sensitivity of the 95% HPD to the variance of the rate prior presents a serious problem for divergence time estimation. This problem is especially important when there are lineage-specific rates, because their impact is not reduced by sampling more loci. This contrasts to gene-specific rates and residual rates. For these classes of rate variation, sampling more loci reduces their impact. This in turn means the assumptions of the rate prior are less likely to be violated and the 95% HPD is more likely to include the correct value (Supplementary Tables S1 and S3 available on Dryad).

A further result from our experiments is that if the variance of the rate prior is less than that of the distribution from which rate variation was simulated, sampling more data (either more loci or more bp at a single locus) can reduce the probability that the 95% HPD includes the correct value. This is most strikingly expressed when there are lineage-specific rates and the unknown divergence time is inferred with a strict clock. In this case, the percentage of experiments that include the correct value within the 95% HPD, falls from 91% when 800 bp or 1 locus is sampled, to 13% or 11.5% when 320,000 bp or 400 loci are sampled (Supplementary Table S3 available on Dryad). Patterns such as this are most clearly observed when there are lineage-specific rates (Supplementary Tables S1 and S3 available on Dryad). This is likely explained by the fact that sampling more data does not reduce the impact of this class of rate variation.

### The implications of lineage-specific rates for divergence time estimation with empirical data sets

Sister taxa comparisons in our *Ipomoea* data set indicated lineage-specific rates that are comparable to the parameters explored in our simulation experiments. Further, we suggest that sister taxa comparisons within a plant genus are likely to underrepresent the extent of lineage-specific rates that occur at broader phylogenetic scales. Given that stronger lineage-specific rates lead to larger errors in divergence time estimates (Fig. 3, Supplementary Table S4 available on Dryad) lineage-specific rates may be even more problematic for divergence time estimation in deeper phylogenies.

It is difficult to determine precisely the implications of lineage-specific rates when inferring divergence times with empirical data sets. Most time-calibrated phylogenies are far more complex than the three or eight-taxon trees used in our simulations. As well as containing more taxa, they typically incorporate temporal assumptions through the implementation of multiple fossil calibrations (Near and Sanderson 2004; Near et al. 2005; Yang and Rannala 2006; Warnock et al. 2011; Magallon et al. 2015; Warnock et al. 2015) and a constant rate birth–death branching process (Yang and Rannala 1997). These assumptions interact directly with inferences of molecular evolutionary rates, which in turn will have complex effects on the distribution of age estimates (Welch and Bromham 2005; Donoghue and Benton 2007; Magallon et al. 2013; Donoghue and Yang 2016). Further, divergence time estimates in empirical data sets may also be affected by model misspecification that is considerably more complex than in the simulation experiments presented here (Gillespie 1991; Sanderson et al. 2004; Duchêne et al. 2014; Kspeka and Phillips 2015; Field et al. 2019).

None of these complexities are likely to ameliorate the basic finding of this article, that in the presence of lineage-specific rates, increasing either the number of sampled loci or sequence length at a single locus has a limited effect in reducing error in divergence time estimates. This finding is concerning when considered in the context of the other assumptions and sources of evidence used in divergence time estimation. For example, variances of UCLN relaxed clocks are often arbitrarily specified, the constant rate birth–death branching process is often likely to be violated, and the fossil record can often be highly incomplete and provide a misleading temporal framework from which to derive fossil calibrations (Smith 2001; Bromham 2006; Donoghue and Yang 2016).

In the future, models that explicitly account for the relationship between certain traits and lineage-specific rates may be used more widely to infer divergence times (Lartillot and Poujol 2011; Ho 2014; Berv and Field 2018). However, it is currently the case that these models are rarely used, and even if they become increasingly easy to implement, they will inevitably remain sensitive to differing interpretations of how traits evolve. An alternative avenue for future progress may be to use gene screening approaches, such that when multilocus data sets are available, only a subset of genes that conform to a strict molecular clock are used for inferring divergence times (Walker et al. 2017; Alfaro et al. 2018; Smith et al. 2018). In an ideal situation, such an approach enables analyses to bypass problems associated with lineage-specific rates. However, it may often be difficult to determine the nature of rate variation in individual genes. This is because individual genes with short sequence lengths may contain insufficient information with which to make inferences about the nature of rate variation, whilst methods to determine the nature of rate variation typically make inferences by analyzing the variance of root to tip lengths in individual gene trees, and as such may overlook rate differences on individual branches. Further, gene screening approaches cause a large amount of data to be overlooked, and the composition of the remaining data may lead to alternative biases. Consequently, although gene screening approaches may be extremely important for future research, there are legitimate uncertainties relating to their implementation, and they are yet to be widely used. Taken together, divergence time estimation therefore remains one of the most challenging inference problems in molecular phylogenetics, regardless of the quantity of molecular sequence data available.

## Supplementary Material

Data available from the Dryad Digital Repository: http://dx.doi.org/10.5061/dryad.m1n6m0m.
